# WMRCA + : a weighted majority rule-based clustering method for cancer subtype prediction using metabolic gene sets

**DOI:** 10.1186/s41065-025-00487-4

**Published:** 2025-07-07

**Authors:** Guojun Liu, Zhaopo Zhu, Yongqiang Xing, Hu Meng, Khyber Shinwari, Ningkun Xiao, Guoqing Liu

**Affiliations:** 1 School of Life Science and Technology, Inner Mongolia University of Science and Technology, Baotou, China; 2 Inner Mongolia Key Laboratory of Life Health and Bioinformatics, Inner Mongolia University of Science and Technology, Baotou, China; 3 Laboratory of Molecular Biology of Microorganisms, Universidade São Francisco, Bragança Paulista, Brazil; 4 Department of Immunochemistry, Institution of Chemical Engineering, Ural Federal University, Yekaterinburg, Russia; 5 Center for Medical Genetics & Hunan Key Laboratory, School of Life Sciences, and National Clinical Research Center for Geriatric Disorders, Department of Geriatrics, Xiangya Hospital, Central South University, Changsha, China

**Keywords:** Metabolic gene sets, Cancer subtypes, Multi-omics, Lipid metabolism

## Abstract

Accurate classification of cancer subtypes plays a pivotal role in advancing precision medicine. In this study, we introduce WMRCA + , a novel clustering approach based on a weighted majority rule that integrates multi-omics data and incorporates metabolic gene sets to robustly determine the optimal number of clusters for tumor subtype identification. WMRCA + evaluates clustering performance using ten internal metrics and offers comprehensive functionalities for data preprocessing and visualization. When applied to The Cancer Genome Atlas (TCGA) lung cancer dataset using lipid metabolism–related gene sets, WMRCA + outperformed widely used clustering algorithms—including iCluster, SNF, NMF, CC, and CNMF—achieving an AUC of 0.947. WMRCA + provides robust, interpretable, and biologically meaningful clustering results, offering a valuable tool for improving the accuracy of cancer subtype prediction. The WMRCA + R package is freely available at https://github.com/guojunliu7/WMRCA.

**Supplementary Information:**

The online version contains supplementary material available at 10.1186/s41065-025-00487-4.

## Highlights

We refined the cluster-of-clusters framework by introducing the Weighted Majority Rule-based Cluster-of-Clusters Approach (WMRCA), enabling accurate tumor subtype prediction through the integration of multi-omics data.

A strong link between metabolic profiles and tumor classification was identified, highlighting the potential of metabolic characteristics as key indicators for distinguishing tumor subtypes.

The WMRCA+ model, incorporating lipid metabolism–related genes, outperformed several widely used clustering algorithms, demonstrating its enhanced predictive power in cancer subtype classification.

**Supplementary Information:**

The online version contains supplementary material available at 10.1186/s41065-025-00487-4.

## Introduction

Cancer remains one of the most challenging diseases faced by humanity. In 2020, there were an estimated 19.3 million new cases of cancer and almost 10.0 million cancer-related deaths worldwide. The most commonly diagnosed cancers were female breast cancer and prostate cancer, while the leading causes of cancer-related death were lung, liver, and stomach cancers [1]. In cancer treatment, balancing therapeutic outcomes with the quality of life for patients continues to pose a significant challenge [2]. Tumor molecular subtyping is the classification of tumors based on molecular analysis techniques, shifting the focus of tumor classification from pathological characteristics to molecular features. This approach helps researchers explore tumor heterogeneity at the molecular level, thereby enabling precision therapies tailored to specific subtypes and ultimately improving patients'quality of life [3–5].

With the growing interest in tumor molecular subtyping, a variety of models and tools have emerged, providing effective support for the prediction and diagnosis of tumor subtypes through diverse technical approaches such as gene expression profiling, pathological image analysis, and deep learning [6, 7]. Several classification algorithms—such as consensus clustering (CC) and non-negative matrix factorization (NMF)—as well as tools like Genefu and OncoNetExplainer, rely on gene expression data and are primarily designed for single-omics applications [8, 9]. NMF, a widely used technique in data mining and machine learning, decomposes a non-negative matrix into the product of two or more smaller non-negative matrices. This approach is particularly useful in processing bioinformatics data with non-negative characteristics, such as gene expression profiles [10]. For instance, Jiang et al. employed NMF to identify three hepatocellular carcinoma subtypes associated with early-stage hepatitis B virus infection [11]. CC, an unsupervised clustering technique, has also been extensively applied in cancer subtype classification [12–15]. For example, Thorsson et al. analyzed pan-cancer data from the TCGA database, integrating high-throughput data across diverse tumor types. Using CC, they classified approximately 10,000 samples from 33 tumor types into six immune subtypes and examined the molecular characteristics of each group [15].

Algorithms that integrate multi-omics data offer greater accuracy in identifying cancer subtypes, laying a solid foundation for personalized treatment strategies. Commonly used clustering methods for multi-omics integration include iCluster, Similarity Network Fusion (SNF), Consensus Non-negative Matrix Factorization (CNMF), and Cluster-of-Clusters Analysis (COCA), all of which have been widely applied in predictive modeling for cancer subtyping. For example, methods such as COCA and iCluster improve clustering robustness by integrating results from multiple clustering algorithms [16]. Specifically, iCluster functions as an ensemble clustering approach, enhancing clustering accuracy by aggregating the outputs of several base clustering methods [17]. CNMF, an extension of NMF, applies matrix factorization techniques to uncover latent structures within high-dimensional data. It distinguishes itself by incorporating constraints such as sparsity, smoothness, and improved interpretability into the factorization process [18]. SNF, by contrast, utilizes spectral theory to efficiently fuse multiple data types and partition samples into distinct clusters [19]. Despite these advancements, achieving precise and robust integration of multi-omics data remains a significant challenge. Enhancing ensemble strategies and incorporating comprehensive evaluation metrics may substantially improve the stability and accuracy of clustering results.

Machine learning has emerged as a powerful approach for classifying and characterizing tumors based on diverse attributes, driving advances in medical diagnostics [20–22]. This approach involves the in-depth analysis of genomic sequences, radiological images, clinical records, and ancillary data to identify subtle cancer subtypes. Such classifications are critical, as they provide essential information for treatment planning and prognostic evaluation [23]. ROAM, a sophisticated transformer-based weakly supervised learning method, is specifically designed for the accurate clinical diagnosis of gliomas and the identification of molecular markers. It excels at extracting complex, multi-scale features from pathological images to enhance diagnostic precision in glioma detection, subtype classification, and biomarker discovery. Notably, ROAM has demonstrated strong performance across both internal and external datasets, including those from The Cancer Genome Atlas (TCGA), highlighting its generalizability and potential as a valuable tool in glioma research and clinical practice [24]. Deep Subspace Mutual Learning (DSML) is another model developed for cancer subtype prediction. By leveraging deep learning and subspace learning techniques, DSML effectively extracts features from high-dimensional data to enable accurate subtype classification [25]. Although these algorithms achieve impressive predictive accuracy, they typically lack the ability to automatically determine the optimal number of clusters. Moreover, automating the classification of cancer patients into distinct molecular subtypes remains a significant challenge. Improving this process could not only reduce time and computational costs, but also enhance diagnostic consistency.

In this study, we developed a weighted majority rule-based clustering algorithm (WMRCA) that integrates multi-omics data—including mRNA, miRNA, lncRNA, DNA methylation, and CNV—for accurate tumor subtype prediction. The model incorporates metabolic gene sets for feature selection, applies a weighted integration strategy across ten evaluation metrics to determine the optimal number of clusters, and supports flexible data preprocessing and visualization. We further introduced WMRCA +, which utilizes a lipid metabolism–related gene set, and demonstrated its superior performance over commonly used clustering methods in cancer subtype classification.

## Materials and methods

### Data sources for WMRCA

All public datasets from the lung adenocarcinoma (LUAD) cohort and lung squamous cell carcinoma (LUSC) cohort in The Cancer Genome Atlas (TCGA) database were downloaded from the UCSC Xena website (https://xenabrowser.net/datapages/). The dataset includes mRNA, miRNA, lncRNA, DNA methylation, copy number variation (CNV), and corresponding clinical files for 1,135 patient samples. The lncRNA and mRNA datasets were extracted from the RNA-Seq data by mapping to the genome reference file (Homo_sapiens.GRCh38.107.chr.gtf.gz) downloaded from the Ensembl Genome Browser (https://asia.ensembl.org/). The metabolic gene sets were derived from previous studies [26, 27]. To enhance the precision of our model, we created a general integrated metabolic pathway gene set by taking the union of the obtained gene sets.

### Data preprocessing and quality control in WMRCA

Missing and very low expression values in the raw data can be filtered out to obtain a high-quality dataset. This filtration reduces computational complexity and enhances both the accuracy and interpretability of clustering models. Therefore, the following preprocessing functions were embedded in the WMRCA model: (1) “data.imputation”, (2) “data.filter”, (3) “data.normalization”, (4) “quantileNormalizeByFeature”, and (5) “cnv2matrix”. The “data.imputation” function offers three optional methods: median filling, mean filling, and K-nearest neighbor filling. The default method is set to K-nearest neighbor filling. The “data.normalization” function primarily includes decentralization and logarithmic transformation, with three normalization options for users to choose from: median, mean, and Z-score. By default, median normalization is applied to ensure a balanced and robust baseline for data scaling.

Next, genes with a high proportion of missing values are filtered out, as such data generally lacks statistical significance and could negatively impact the accuracy of the results. The “data.filter” function first calculates the number of missing values for each gene across all samples, divides this number by the total number of samples, and then filters the data based on a pre-set threshold (default is 0.8). When datasets come from different platforms (e.g., microarray and RNA-Seq), Franks et al. recommend using feature-specific quantile normalization (FSQN) for more accurate cancer subtype classification [28]. For FSQN, we used the “quantileNormalizeByFeature” function from the FSQN package (https://github.com/jenniferfranks/FSQN). Segment-based copy number variation data typically includes sample ID, chromosome, start and end positions, and calculated values, but cannot be directly analyzed for clustering. However, we have developed the “cnv2matrix” function, which converts copy number variation data into a matrix format, with genes as rows and samples as columns.

### Feature selection for WMRCA

The WMRCA framework provides custom feature selection for various omics data types, with the goal of identifying the optimal subset of features for downstream analysis. The feature selection step is optional and can precede clustering. We implemented four methods for this step: metabolism-based selection, variance, Principal Component Analysis (PCA), and Median Absolute Deviation (MAD). Notably, variance, PCA, and MAD are commonly used in cancer genomics studies due to their effectiveness in identifying informative features. Furthermore, the use of metabolomics datasets facilitates the identification of metabolic subtypes, which reflect the metabolic reprogramming frequently observed in cancer.

### Principle of the WMRCA model

The method described in our previous study [11] was used to perform COCA on mRNA, miRNA, lncRNA, DNA methylation, and CNV data, which resulted in the identification of molecular subtypes ${C}_{m}$ for the ${m}^{th}$ omics data type. The cluster assignment of sample *i* in omics data *m* is represented by a binary vector ${V}_{im}$ of length ${C}_{m}$. Based on the CC method proposed by Monti et al. [29], the resulting binary matrix ${B}_{NC}$ was clustered to determine the final sample-level subtypes, where *N* is the number of samples and *C* is the total number of derived molecular subtypes. The definitions of the binary vector ${V}_{im}$ and the number of subtypes *C* are provided below:

1 $${\text{V}}_{\text{im}} \left(\text{j}\right) = \left\{\begin{array}{cc}1& \text{if i belongs to cluster j}\\ 0& \text{otherwise}\end{array}\right.$$

2 $$C = \sum_{i=1}^{M}{C}_{m}$$

The assessment of the optimal number of clusters is performed using ten internal unsupervised clustering evaluation indices, and the results are visualized accordingly. Eight indices—including the mean silhouette, the Calinski-Harabasz index, the entropy, the Dunn index, the Dunn index 2, the within-between SS ratio, the Pearson gamma, and the gap statistic—are calculated using the “fpc” R package [30]. In addition, two widely used internal evaluation indices have been incorporated into the assessment: the proportion of ambiguous clustering (PAC) and the mean cophenetic distance [31, 32]. PAC is derived from the cumulative distribution function (CDF) curve. The definitions of CDF, PAC, and the optimal number of clusters *K* are as follows:

3 $$CDF\left(c\right) = \frac{{\sum }_{i<j}I\left\{M \left(i,j\right) \le c\right\}}{N \left(N-1\right)/2}$$

4 $${PAC}_{k} \left({x}_{1},{x}_{2}\right) = {CDF}_{k}\left({x}_{2}\right) - {CDF}_{k} \left({x}_{1}\right)$$

5 $$\mathrm{optimal}\;K=\arg\;\underset k{\text{min}}{\;PAC}_k$$

Let *M(i,j)* denote the consensus matrix, *I*{⋅} the indicator function, *N* the number of rows in *M*, and *c* the consensus index value; *i*(*j*) represents the sample identifier. The PAC is defined as the proportion of sample pairs for which the consensus index value falls within the interval (${x}_{1}$, ${x}_{2}$)  ⊂ [0,1], where ${x}_{1}$ is a value close to 0 and ${x}_{2}$ is a value close to 1. A lower PAC value indicates a flatter middle section in the cumulative distribution function, suggesting a lower rate of inconsistent clustering assignments. Therefore, the optimal number of clusters can be inferred by identifying the *K* value corresponding to the lowest PAC. The mean cophenetic distance is a metric that reflects the dispersion of the consensus matrix. It is calculated based on the Pearson correlation coefficient between the original distance matrix—such as Pearson, Kendall, or Spearman—and the consensus matrix, which is typically obtained from a ConsensusClusterPlus run [33]. The closer the correlation coefficient is to 1, the more stable and suitable the clustering solution. The definition of the mean cophenetic distance is as follows:

6 $$\text{Mean Cophenetic Distance} = \frac{1}{\left(\begin{array}{c}n\\ 2\end{array}\right)} \sum_{i=1}^{n-1}\sum_{j=1+1}^{n}{d}_{ij}$$

Here, *n* denotes the number of samples, ${d}_{ij}$ represents the cophenetic distance between samples *i* and *j*, and $\left(\genfrac{}{}{0pt}{}{n}{2}\right)$ indicates the total number of possible combinations for selecting two samples from *n* samples. Because different evaluation indices may exhibit varying levels of bias when applied to different omics datasets, we adopt a weighted majority voting rule to obtain more reliable results. Let ${d}_{t,j}$∈{0, 1} denote the decision of the ${t}^{th}$ index, where *t* = 1,…,*T* and *j* = 1,…,*C*, with *T* being the number of indices and *C* the number of classes. If the ${t}^{th}$ classifier selects class *j*, then ${d}_{(t,j)}$= 1; otherwise, ${d}_{(t,j)}$= 0 [34]. In weighted voting, each classifier ${d}_{(t,j)}$ is assigned a weight ${w}_{t}$, and the predicted class label for class *j* is calculated as follows:

7 $$\mathrm{Prediction}\left(j\right)=\left\{\begin{array}{cc}1&if\sum_tw_td_{t,j}\geq\theta\\0&\text{otherwise}\end{array}\right.$$

8 $$J ={\text{argmax}}_{j\in \left\{\text{1,2},\cdots ,C\right\}}\sum_{t=1}^{T}{w}_{t}{d}_{t,j}$$

For class *j*, the sum $\sum_{t=1}^{T}{w}_{t}{d}_{t,j}$ represents the total votes for class *j*. The weight ${w}_{t}$ can either remain constant throughout the process—determined by experts or through offline training—or vary over time. The *θ* is an appropriate threshold, ranging from 0 to 1. For example, when *θ* is set to 1 and the weights ${w}_{t}$ of two specific indices are set to 0.5, each of these indices contributes a maximum of 0.5 to the vote. In this case, a prediction for class *j* is 1 if both indices vote for the same class, reaching the threshold *θ*. WMRCA provides hierarchical clustering, K-means, and centroid-based partitioning as clustering algorithm options, with hierarchical clustering being set as the default algorithm due to its relatively higher accuracy. In this study, ten indices were used to evaluate the clustering performance, so *T* was set to 10. For simplicity, both *θ* and the weights ${w}_{t}$ were fixed at 1.

### Reliability evaluation of the WMRCA

Five indices are used to quantify predictive performance: sensitivity (${S}_{n}$), specificity (${S}_{p}$), positive predictive value (*PPV*), negative predictive value (*NPV*), and accuracy (*ACC*). These metrics are defined as follows:

9 $${S}_{n} =TP/\left(TP +FN\right)$$

10 $${S}_{p} =TN/\left(TN +FP\right)$$

11 $$PPV = \frac{TP}{TP +FP}$$

12 $$NPV = \frac{TN}{TN +FN}$$

13 $$ACC = \frac{TP+TN}{TP +TN+FP+FN}$$

The terms *TP*, *FN*, *TN*, and *FP* respectively represent the number of true positives, false negatives, true negatives, and false positives. The quality of the proposed method is further evaluated by calculating the area under the receiver operating characteristic curve (AUC). To investigate differences in lipid metabolic pathways between subtypes, gene set variation analysis is conducted using the R package “GSVA” [35], and the results are visualized with the “pheatmap” package [36]. Clinical differences between subtypes were assessed using the Chi-square test, and immune cell infiltration levels were estimated using CIBERSORT, a web-based tool for deconvoluting immune cell composition from bulk gene expression profiles [37].

## Results

### Data acquisition

The non-small cell lung cancer (NSCLC) dataset downloaded from the public TCGA database was divided into two cohorts: one consisting of patients with lung adenocarcinoma (LUAD), which is characterized by a high mutation rate, and the other consisting of patients with lung squamous cell carcinoma (LUSC), which has a lower mutation rate, totaling 1,027 cases (526 LUAD and 501 LUSC). Multi-omics data, including mRNA expression, miRNA expression, lncRNA expression, copy number variation, and DNA methylation, along with the clinical information of NSCLC patients, were collected. A comparison of the number of features before and after data filtering is summarized in Table 1. The distributions of the mean, variance, and median absolute deviation of features before and after filtering are shown in Figures S1 and S2, respectively.

**Table 1:** Comparison of feature counts before and after data filtering

	*Dataset*	*Before filtering*	*After filtering*	*Sample size*
NSCLC	mRNA	19229	1000	1027
	miRNA	1881	800	994
	lncRNA	9907	1000	1027
	CNV	24776	1000	1017
	Methylation	485577	1000	830

The feature filtering process encompasses data preprocessing and feature selection steps. The sample size refers to the number of patients diagnosed with NSCLC (Non-Small Cell Lung Cancer)

### Workflow of WMRCA

**Fig. 1:**
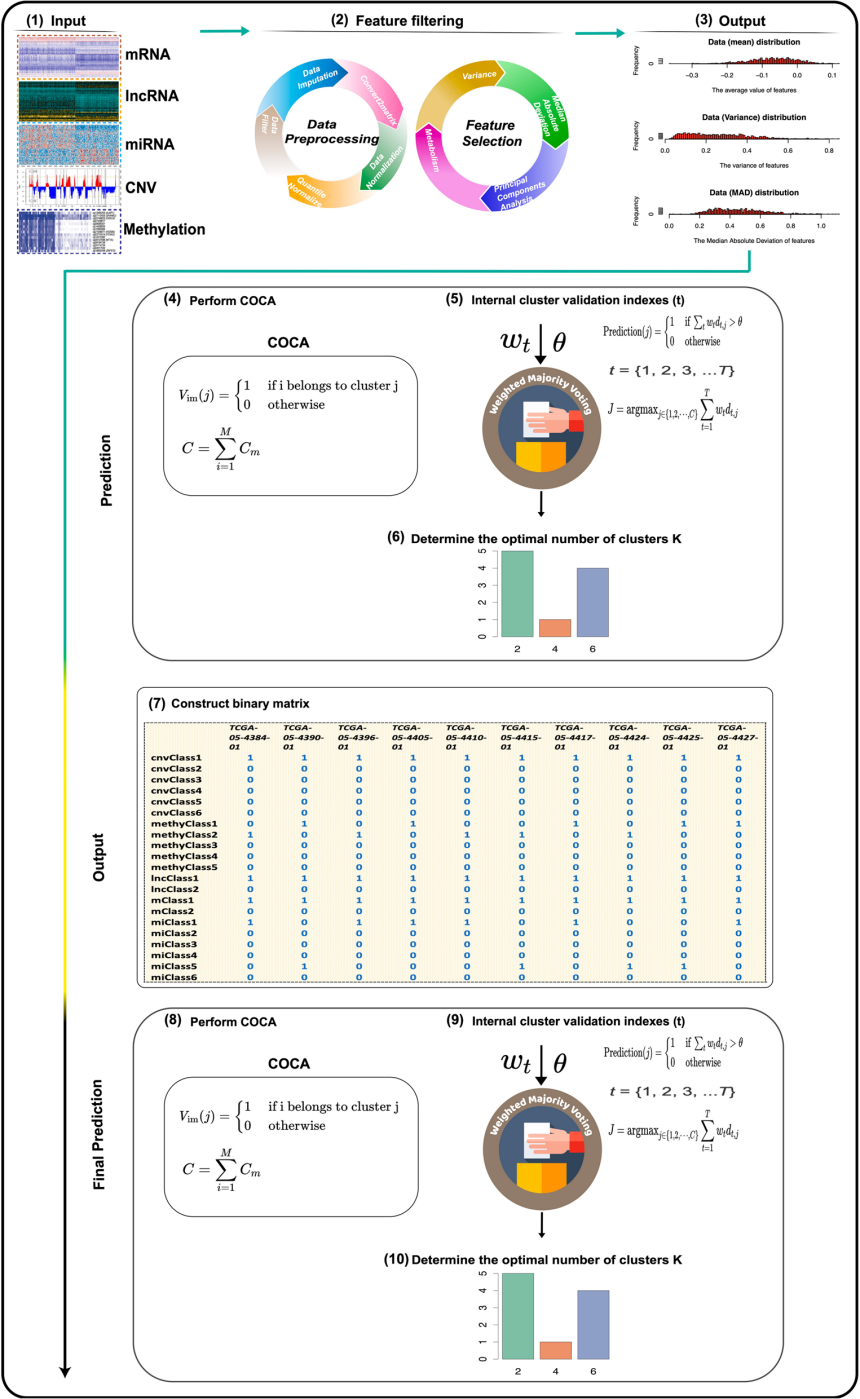
Workflow diagram of the WMRCA model

The foundation of the WMRCA model lies in CC algorithms, which require the selection of appropriate distance and linkage methods. The “ConsensusClusterPlus” function, embedded in the WMRCA framework, uses “hc” for clusterAlg, “pearson” for distance, 6 for *K*, and “complete” for innerLinkage as default values. The WMRCA workflow, illustrated in Fig. 1, begins with five datasets as input and generates high-quality data through a series of preprocessing and feature selection steps. The integration of consistent clustering results from mRNA, miRNA, lncRNA, CNV, and methylation data was achieved using COCA. The optimal number of clusters, *K*, was determined based on a weighted majority voting rule. A binary or relational matrix was then constructed using the selected K, followed by another round of COCA and weighted majority voting to generate the final clustering result. This algorithm retains the simplicity of the CC approach: users only need to input the mRNA, miRNA, lncRNA, CNV, and methylation data, configure the relevant parameters, and complete clustering in a single step to obtain the optimal number of subtypes. The integration of multi-omics data, combined with repeated application of COCA and weighted majority voting, produced clustering results that are theoretically more accurate and robust than those generated from single-omics data.

### Determination of the optimal number of clusters using consensus clustering

**Fig. 2:**
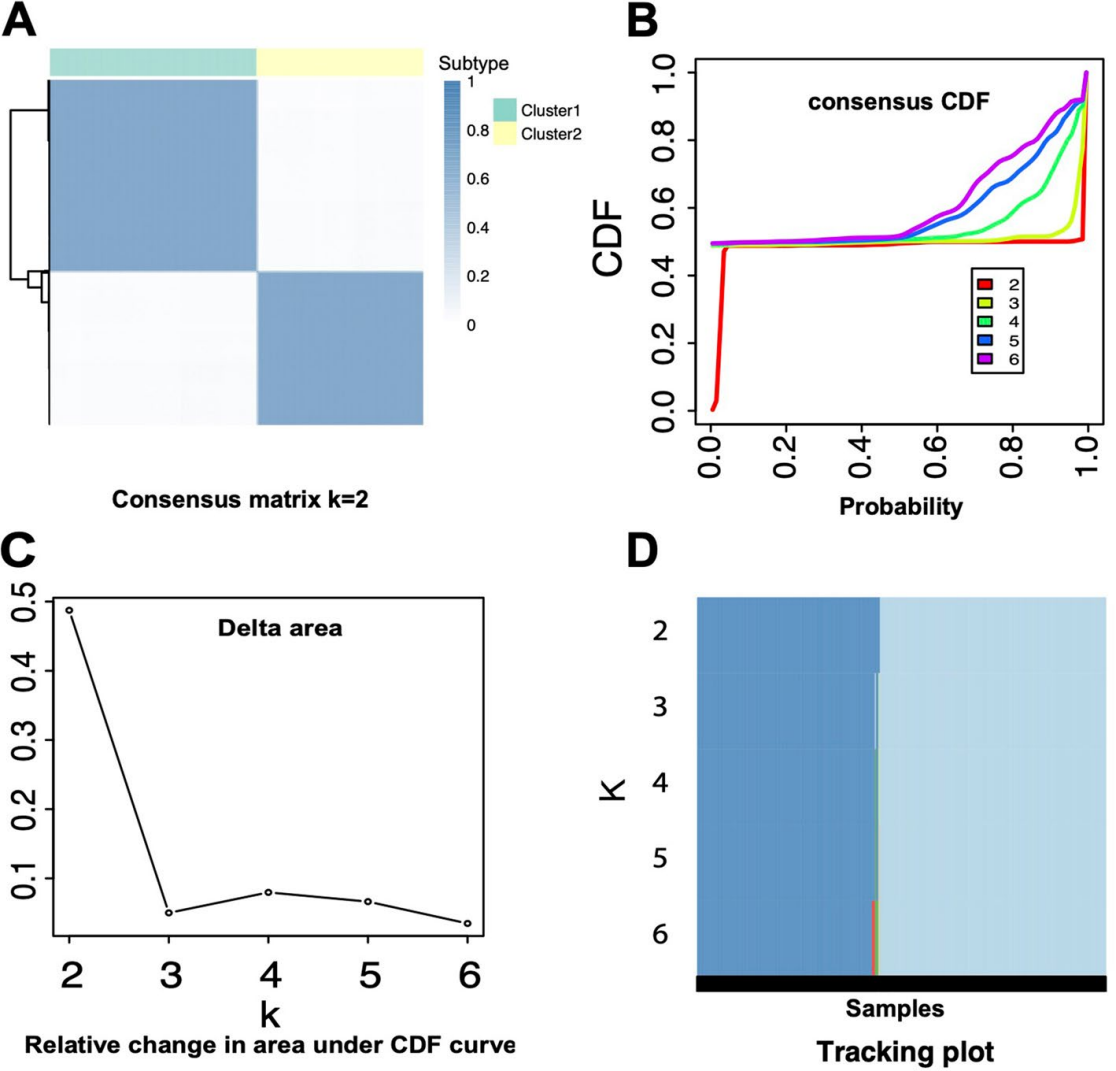
Determination of optimal cluster number using ConsensusClusterPlus. **A** Consensus matrix heatmap for K = 2, showing high intra-cluster consensus. **B** Cumulative distribution function (CDF) plot, where K = 2 yielded the flattest curve, indicating stable clustering. **C** Relative change in the area under the CDF curve across different K values, with the inflection point at K = 3 suggesting a plateau in clustering improvement. **D** Tracking plot displaying sample assignments across K = 2 to K = 6; the minimal sample instability observed at K = 4 supports it as the most stable solution

The heatmap was generated using the “ConsensusClusterPlus” R package based on the consensus matrix for *K* = 2 and is presented in Fig. 2A. In the consensus cumulative distribution function (CDF) plot, the optimal condition is characterized by a sharp increase in the curve at values close to 0 and 1. Under these conditions, the CDF reached an approximate maximum at *K* = 2, indicating the most reliable clustering results (Fig. 2B). The comparison of the relative change in the area under the CDF curve between *K* and *K*–1 is illustrated in Fig. 2C. Since there is no value for *K* = 1, the result at *K* = 2 represents the total area under the CDF curve. This trend suggested that at *K* = 3—corresponding to the elbow position—the area tended to stabilize, thus indicating that the optimal number of clusters is 3. The tracking plot in Fig. 2D shows individual samples as black bars, representing their cluster assignments across various *K* values. Each colored segment represents a distinct cluster. Frequent changes in color across different K values reflect unstable classifications. In this analysis, the transition from *K* = 4 to *K* = 5 resulted in the fewest unstable samples—only one—suggesting the most stable clustering at *K* = 4. Therefore, the tracking plot indicated that *K* = 4 is the optimal number of clusters. The results from the ConsensusClusterPlus package demonstrated that *K* = 2, *K* = 3, and *K* = 4 were all plausible candidates for the optimal number of clusters. This outcome highlights a limitation of traditional CC analysis, which often struggles to determine a clearly optimal cluster number when predicting cancer subtypes.

### Enhancing subtype prediction through metabolic pathway profiling

Cancer cells differ significantly from normal cells in certain metabolic pathways. In general, they undergo metabolic reprogramming and exhibit alterations in the immune microenvironment [38]. Therefore, metabolic pathway screening is essential for predicting specific cancer subtypes. To support this objective, the WMRCA package includes a metabolic pathway screening function, which allows users to identify and analyze genes involved in relevant pathways. The WMRCA model utilizes gene sets related to seven types of metabolism: lipids, carbohydrates, amino acids, energy, nucleotides, vitamins and cofactors, and the tricarboxylic acid (TCA) cycle. Table 2 presents the number of genes associated with each metabolic pathway category. Expression profiles of 1,390 genes related to lipid metabolism were specifically extracted for further analysis. The term WMRCA + indicates an extended version of the WMRCA model that emphasizes lipid metabolism genes in patient subtype classification. This refined strategy is designed to improve the accuracy of cancer subtype prediction by incorporating a more comprehensive view of metabolic pathway activity.

**Table 2:** Description of each metabolic pathway dataset used in WMRCA

*Metabolic pathway*	*Associated genes*
Lipid	1390
Carbohydrate	1314
Amino acid	657
Energy	410
Nucleotide	236
Vitamin and cofactor	168
TCA cycle	149

### *Evaluation of clustering performance and subtype determination using WMRCA and WMRCA* + 

**Fig. 3:**
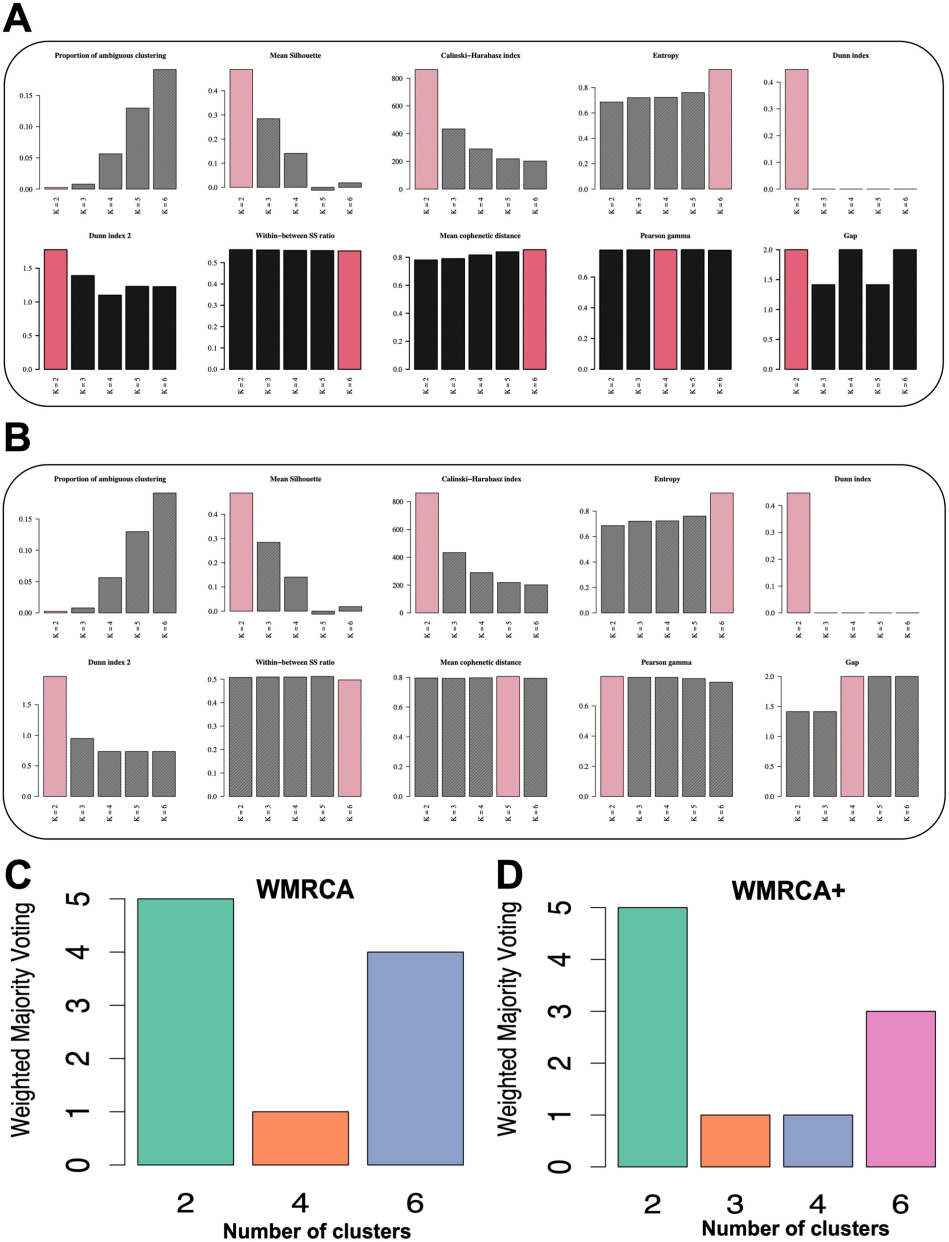
Ten indices and the weighted majority voting method automatically obtain the optimal number of clusters. **A** Indices used in WMRCA. **B** Indices used in WMRCA +. **C** Weighted majority voting method for WMRCA. **D** Weighted majority voting method for WMRCA +

The WMRCA model evaluated clustering performance across values of *K* ranging from 2 to 6 using ten indicators: the proportion of ambiguous clustering, mean cophenetic distance, mean silhouette score, Calinski–Harabasz index, entropy, Dunn index, Dunn index 2, within–between sum-of-squares ratio, Pearson gamma, and the gap statistic. Depending on the specific indicator, either the highest or lowest value—highlighted by red bars in the bar chart—reflected the best sample-to-subtype match (Fig. 3A). The WMRCA + model applied the same ten indicators to assess clustering results across the same *K* range (Fig. 3B). The weighted majority voting method automatically integrated output from five omics datasets and ten clustering evaluation algorithms. In this analysis, weighted majority voting was conducted six times: once for each omics dataset (mRNA, miRNA, lncRNA, CNV, and methylation), and once again on the binary matrix generated by Eq. (1) to finalize the number of clusters. Regardless of whether WMRCA or WMRCA + was used, lung cancer samples from the TCGA database were consistently classified into two major subtypes, with six subtypes identified as suboptimal (Fig. 3C, D). Since the ten algorithms have inherent data preferences, we recommend initially setting both *θ* and weights ${w}_{t}$ to 1 to 1. Based on the results, we can then decide whether to adjust the weight of specific indices. If a particular index demonstrates better performance for the data, its weight can be increased, thus controlling the diversity of the majority voting.

### Comparison of cancer subtype discovery methods and classification accuracy

**Fig. 4:**
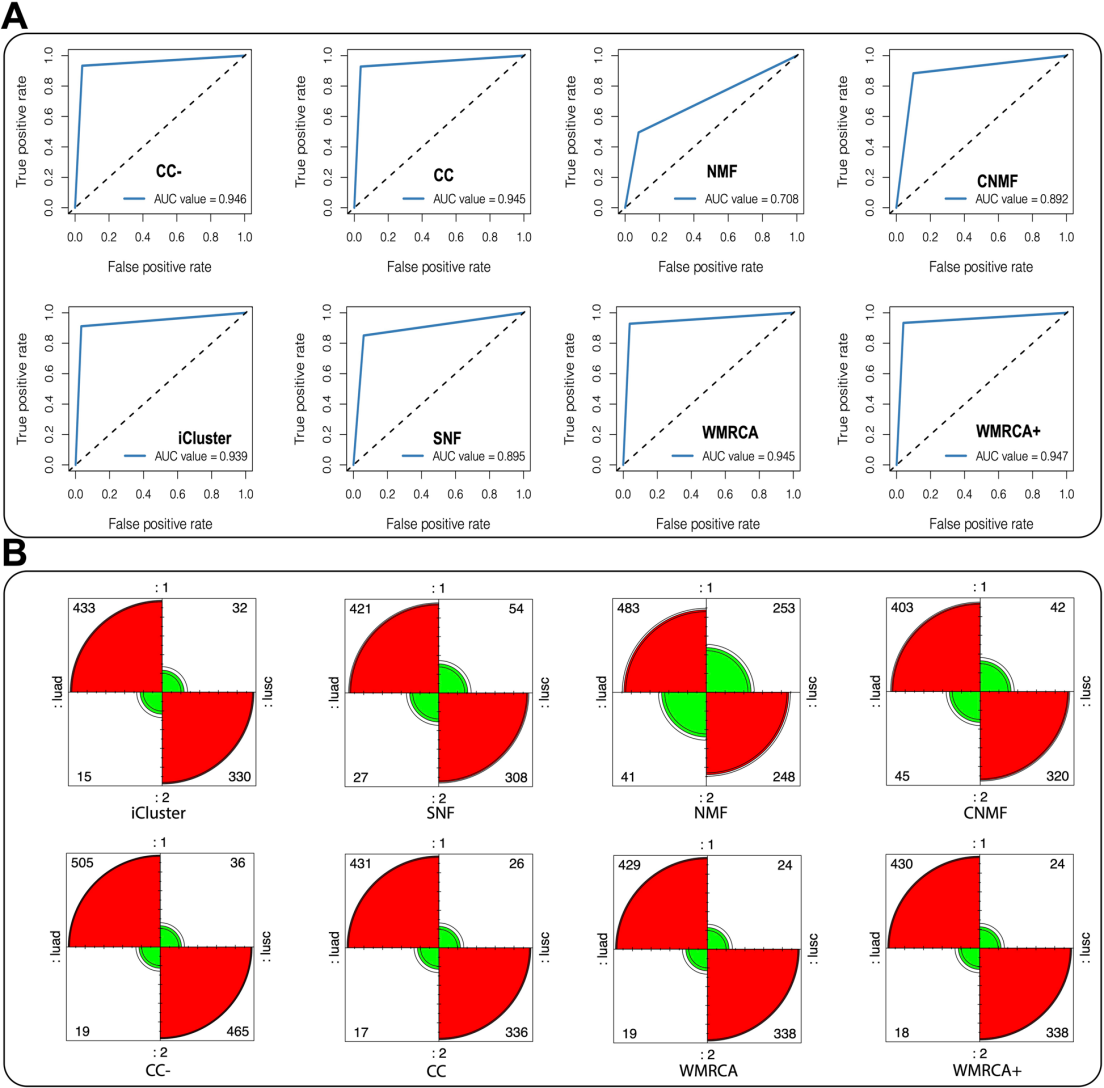
Performance comparison of clustering algorithms based on ROC curves and confusion matrices. **A** Receiver Operating Characteristic (ROC) curves for various clustering algorithms, illustrating their classification performance in predicting cancer subtypes. Higher AUC values indicate better predictive accuracy. **B** Confusion matrix results for each algorithm, providing a visual summary of classification outcomes, including true positives, false positives, true negatives, and false negatives

Performance comparisons among several widely used cancer subtype discovery methods revealed that, based on five evaluation indicators, WMRCA + achieved the best performance, followed by WMRCA, iCluster, and CC, in that order. It is important to note that CC and NMF are specifically designed for single-omics datasets, such as gene expression data, whereas iCluster, SNF, CNMF, and WMRCA are applicable to multi-omics data analysis. Moreover, both WMRCA + and WMRCA support the automatic selection of the optimal number of clusters (*K*) (Table 3). The receiver operating characteristic (ROC) curve further demonstrated that WMRCA +, CC, WMRCA, and iCluster outperformed other algorithms, with respective AUC values of 0.947, 0.946, 0.945, and 0.945. This result highlighted the superior classification accuracy of models incorporating lipid metabolism genes as key features (Fig. 4A). The use of a confusion matrix enabled a detailed evaluation of subtype prediction performance. In this matrix, ‘luad’ and ‘lusc’ represented the actual observed categories, while ‘1’ and ‘2’ indicated the predicted subtypes, providing a direct and quantitative measure of model accuracy (Fig. 4B).

**Table 3:** Performance comparison of WMRCA in NSCLC subtype clustering

	*S*_*n*_	*S*_*p*_	*PPV*	*NPV*	*ACC*	*MOS*	*ASONC*
iCluster	0.912	0.967	0.957	0.931	0.942	*Y*	*N*
SNF	0.851	0.94	0.919	0.886	0.9	*Y*	*N*
NMF	0.495	0.922	0.858	0.656	0.713	*N*	*N*
CNMF	0.884	0.9	0.877	0.906	0.893	*Y*	*N*
CC-	0.928	0.964	0.961	0.933	0.946	*N*	*N*
CC	0.928	0.962	0.952	0.943	0.947	*Y*	*N*
WMRCA	0.934	0.958	0.947	0.947	0.947	*Y*	*Y*
WMRCA +	0.934	0.96	0.949	0.947	0.948	*Y*	*Y*

WMRCA + not only performs the same data preprocessing as WMRCA but also conducts feature selection based on lipid metabolism gene sets. CC- indicates Consensus Clustering performed using a single mRNA dataset.'Y'stands for'yes,'and'N'stands for'no.'ASONC refers to the automatic selection of the optimal number of clusters. MOS indicates multi-omics support

### Clinical indicators, prognostic analysis, and immune infiltration in LUAD and LUSC

**Fig. 5:**
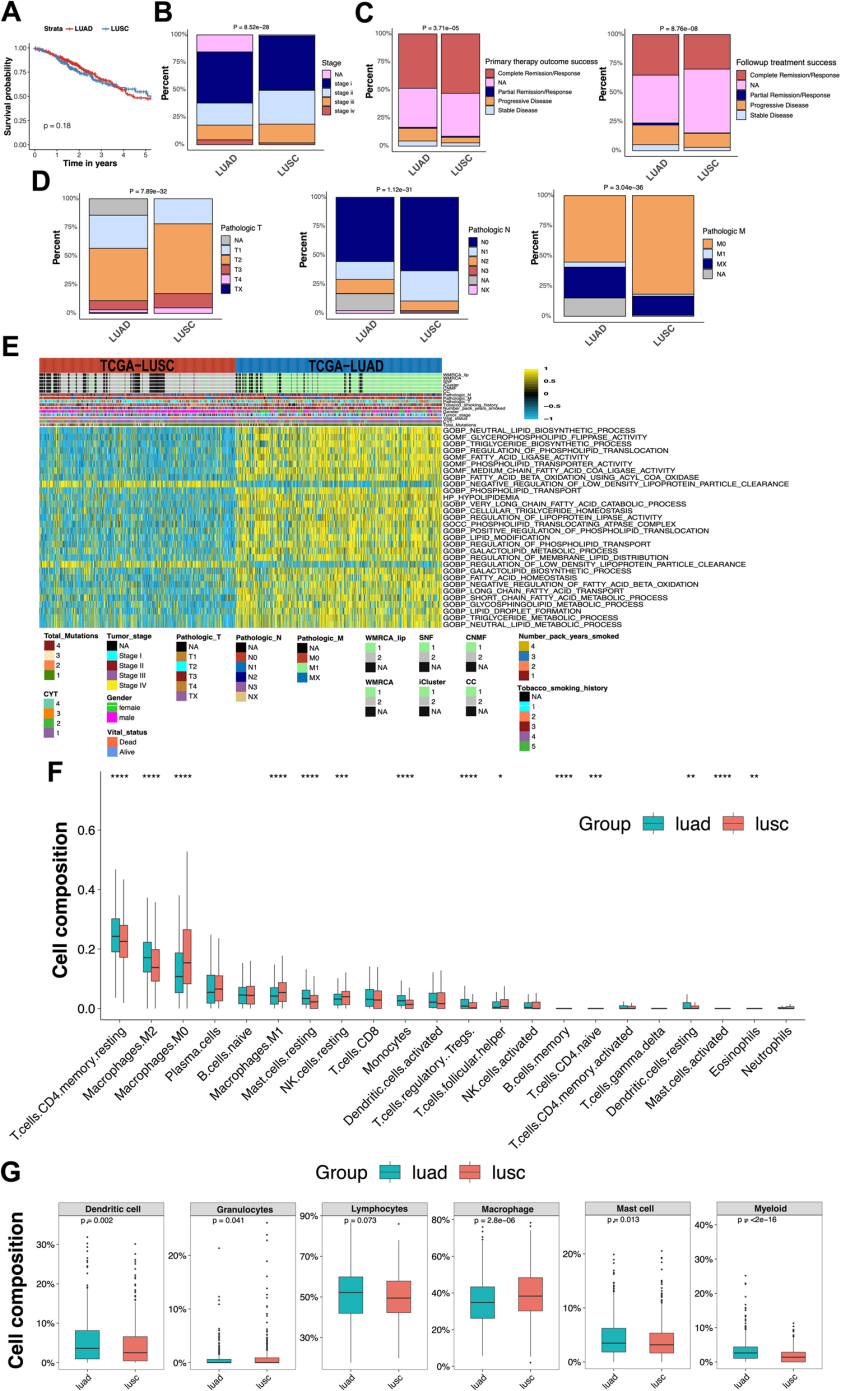
Comparative clinical, pathological, metabolic, and immunological characteristics of LUAD and LUSC patients. **A** Kaplan–Meier survival analysis revealed no statistically significant difference in overall survival between LUAD and LUSC patients (log-rank test, *P* = 0.18). **B** Bar plot comparing clinical stage distributions at diagnosis, showing that LUAD tends to be diagnosed at more advanced stages, while LUSC is often detected earlier. **C** Comparison of primary therapy outcomes and follow-up treatment outcomes after neoadjuvant treatment. **D** TNM staging comparisons between LUAD and LUSC. **E** Heatmap based on Gene Set Variation Analysis (GSVA) depicting 30 significantly enriched lipid metabolism pathways (*P* < 0.05). **F** Infiltration analysis of 22 individual immune cell types revealed distinct immune landscapes between LUAD and LUSC. **G** Aggregated infiltration profiles of six major immune cell categories, further emphasizing subtype-specific immune microenvironment differences

Further prognosis analysis of LUAD and LUSC patients was performed using the Kaplan–Meier plot method. As shown in Fig. 5A, no significant difference in survival times was observed between the two groups (Log-rank test, *P* = 0.18). LUAD is more likely to be overlooked in its early stages and progress to advanced stage IV. In contrast, LUSC, due to its more noticeable symptoms, is often detected earlier, sometimes even before stage II (Fig. 5B). Regarding primary therapy outcomes, LUSC patients showed a significantly higher proportion of complete and partial responses after neoadjuvant therapy compared to LUAD. In contrast, LUAD patients demonstrated less effective outcomes after neoadjuvant therapy, with a significantly higher proportion of progressive and stable disease compared to LUSC (Fig. 5C). The TNM system stages tumors based on size, lymph node involvement, and distant metastasis. As shown in Fig. 5D, LUSC tumors exhibit higher levels of tumor size and local extension (T), lymph node involvement (N), and distant metastasis (M) compared to LUAD. Notably, both LUAD and LUSC subtypes contain a substantial number of samples with NA values. Therefore, we excluded these samples, and the chi-square test revealed statistically significant differences in clinical indicators between LUAD and LUSC, including pathological T, pathological N, pathological M, tumor stage, age at diagnosis, gender, pack-years smoked, smoking history, and primary therapy outcome success (Table S1). Subsequently, gene set variation analysis (GSVA) was conducted, and a heatmap was generated with a p-value threshold of 0.05 to identify 30 significantly enriched lipid metabolism pathways. In LUAD, processes such as triglyceride biosynthesis, fatty acid beta-oxidation, and lipid droplet formation were intensified, indicating that LUAD relies more heavily on lipid metabolism compared to LUSC (Fig. 5E). Additionally, the fatty acid biosynthesis process was enhanced in LUAD, potentially leading to increased synthesis of complex lipids, such as phospholipids and glycosphingolipids, suggesting augmented de novo lipid synthesis in LUAD (File S1). Dendritic cells, lymphocytes, mast cells, and myeloid cells showed higher infiltration levels in LUAD, whereas granulocytes and macrophages exhibited higher infiltration levels in LUSC. These differences in immune cell infiltration patterns may influence the tumor's immune evasion mechanisms, prognosis, and response to immunotherapy (Fig. 5F, G).

### Integrated clustering and comprehensive analysis with WMRCA

**Fig. 6:**
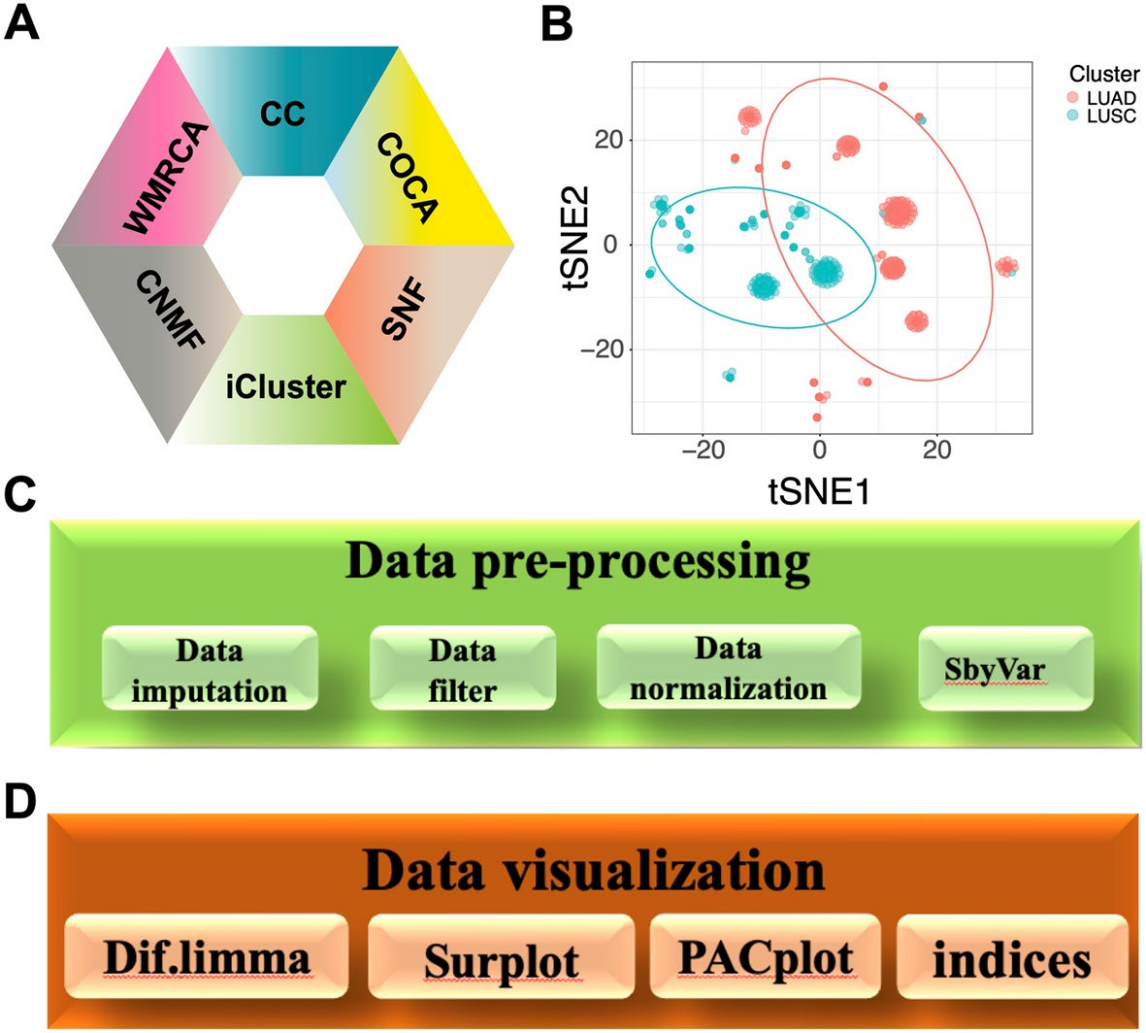
Overview of the WMRCA package and its integrated functionalities. **A** The WMRCA package integrates the WMRCA model with widely used clustering algorithms, including CC, COCA, SNF, iCluster, and CNMF. **B** The WMRCA model employs t-distributed Stochastic Neighbor Embedding (t-SNE) for dimensionality reduction and visualization. **C** The package provides essential preprocessing functions: ‘data.filter’, ‘data.imputation’, ‘SbyVar’, and ‘data.normalization’. **D** Additional utilities include ‘Dif.limma’ for differential expression analysis, ‘Surplot’ for Kaplan–Meier survival visualization, ‘indices’ for internal metric evaluation and weighted voting, and ‘PACplot’ for PCA-based analysis

The WMRCA package integrates the WMRCA model with widely used clustering algorithms such as CC, COCA, SNF, iCluster, and CNMF, positioning it as one of the most comprehensive clustering algorithm packages (Fig. 6A). The WMRCA model employs t-distributed Stochastic Neighbor Embedding (t-SNE) to visually highlight disparities among samples, thereby revealing intrinsic variations (Fig. 6B). In addition to supporting clustering, the WMRCA package includes essential bioinformatics tools for data preprocessing and visualization. Preprocessing functions include ‘data.filter’, ‘data.imputation’, ‘SbyVar’, and ‘data.normalization’, which support key steps such as filtering, missing value imputation, variable selection, and normalization (Fig. 6C). The function ‘Dif.limma’ enables differential expression analysis and generates volcano plots, while ‘Surplot’ performs Kaplan–Meier survival analysis and visualization. The ‘indices’ function calculates ten robust internal evaluation metrics and applies weighted majority voting to assess clustering performance. The ‘PACplot’ function also provides principal component analysis (PCA), collectively enhancing the accuracy and robustness of subtype identification (Fig. 6D).

## Discussion

Unsupervised clustering of multi-omics data and the definition of subtypes remain challenging tasks that demand both methodological rigor and biological relevance. In cancer subtype classification studies, Consensus Clustering (CC) plays a pivotal role [39–41]. Researchers have applied CC with the PAM50 gene set as features to categorize molecular subtypes of breast cancer based on expression profiles, dividing them into luminal, triple-negative, and Her2-positive subtypes, and have developed corresponding treatment strategies based on these classifications [42]. Cluster-of-Clusters Analysis (COCA), an innovative extension of the CC approach, utilizes multi-omics datasets to classify samples into distinct subtypes, potentially uncovering novel disease categories [43–46]. However, the use of only one or a few evaluation algorithms proves insufficient for achieving the accuracy and reliability required for precise clustering outcomes. Specifically, even in real datasets with well-separated clusters, neither the CDF plot nor the relative change in the area under the CDF curve consistently determines the true value of *K*, making it difficult to employ these metrics as reliable benchmarks for class discovery methods [31]. To overcome these limitations, we developed an integrated model that incorporates multi-omics data for the high-precision identification of tumor subtypes.

By comparing various cancer subtype prediction methods, we found that the WMRCA model determines the optimal number of clusters (*K*) with superior predictive accuracy compared to state-of-the-art methods. Multi-omics-based methods generally outperform single-omics-based ones in effectiveness. The WMRCA + model, incorporating feature selection from lipid metabolism genomics, stands out for its enhanced predictive capabilities in cancer subtype classification. Metabolic reprogramming is a hallmark of cancer, with tumor cells adapting through lipid, glucose, and amino acid metabolism [47, 48]. The metabolic heterogeneity across subtypes can contribute to the development of distinct subtypes [49]. Our previous pan-cancer studies showed that de novo fatty acid synthesis is enhanced in most cancers, while cholesterol and triglyceride catabolism is downregulated [50]. Greater lipid and lipid droplet formation correlates with higher tumor malignancy [51]. While we observed enhanced triglyceride biosynthesis, fatty acid β-oxidation, lipid droplet formation, and fatty acid biosynthesis in LUAD, Kaplan–Meier survival analysis did not show significant prognosis differences between LUAD and LUSC. This suggests a more complex relationship between lipid metabolism and tumor malignancy. Clinically, LUAD is often diagnosed at a later stage due to its subtle early symptoms, while LUSC is detected earlier due to more overt symptoms. This difference may explain the disparity in treatment responses: LUSC patients exhibit higher rates of complete and partial responses to neoadjuvant therapy, whereas LUAD patients show more progression or stability post-treatment. According to TNM staging, LUSC tumors have greater size, lymph node involvement, and distant metastasis than LUAD, indicating distinct tumor progression patterns. Immune cell infiltration profiles also differ: LUAD has higher levels of dendritic cells, lymphocytes, mast cells, and myeloid cells, while LUSC is characterized by more granulocytes and macrophages. These immune microenvironment differences likely affect tumor immune evasion, clinical outcomes, and immunotherapy responses. In summary, while subtype-specific therapies hold significant potential, challenges such as tumor heterogeneity, immune evasion, metabolic differences, inconsistent therapeutic targets, and subtype switching remain. Future research must focus on accounting for tumor heterogeneity and dynamic changes in precision medicine.

The WMRCA model stands out for its comprehensive integration of multi-omics data, including genomics, transcriptomics, and epigenomics. It integrates ten robust internal evaluation metrics and applies the weighted majority voting algorithm to dynamically determine the optimal number of clusters. In addition to advanced visualization features, the model offers essential data preprocessing functions, ensuring seamless compatibility with TCGA datasets. In our forthcoming research, we plan to improve the precision of cancer subtype prediction by systematically assessing the contribution of each omics data type. Assigning differential weights to datasets may further improve predictive accuracy. We also intend to investigate the role of lipid metabolism in cancer subtype transformation, focusing on whether levels of key lipid metabolism markers are associated with subtype shifts. Recent studies have shown that LKB1 deficiency promotes tumor plasticity and facilitates the trans-differentiation of lung adenocarcinoma into squamous cell carcinoma in murine models [52]. A deeper analysis of lipid metabolic reprogramming and the tumor microenvironment may offer new insights into the multifaceted role of lipid metabolism in cancer biology. This improved understanding will support the development of more accurate and effective predictive models in oncology. Furthermore, we aim to enhance the algorithm by integrating cutting-edge techniques from deep learning and natural language processing, including deep neural networks [53, 54], multi-view subspace clustering [55], large language models [56], and transformer architectures [57]. These emerging methods have demonstrated increasing value in subtype identification and feature gene prediction.

In a nutshell, accurate prediction of cancer subtypes is essential for effective diagnosis and treatment, and represents a major challenge in precision medicine. The WMRCA + model is positioned to become a powerful tool for oncology professionals, as it supports the creation of personalized treatment strategies and improves therapeutic outcomes. In addition, the model addresses cancer heterogeneity from a metabolic perspective, which enables deeper exploration of the biological mechanisms that drive tumor diversity.

## Supplementary Information

Supplementary Material 1: Figure S1. Distribution of the mean, variance, and median absolute deviation of features before data filtering.

Supplementary Material 2: Figure S2. Distribution of the mean, variance, and median absolute deviation of features after data filtering.

Supplementary Material 3: Table S1. Associations between tumor subtypes and clinical indicators based on chi-square tests.

Supplementary Material 4: File S1. Results of gene set variation analysis for LUAD and LUSC subtypes.

## Data Availability

No datasets were generated or analysed during the current study.

## Abbreviations

WMRCA: Weighted Majority rule-based Cluster-of-Clusters Analysis
NMF, Non-negative Matrix Factorization; TCGA, The Cancer Genome Atlas Program; COCA: Cluster-of-Clusters Analysis
CC: Consensus clustering
CNMF: Consensus Non-negative Matrix Factorization
SNF: Similarity network fusion
NMF: Non-negative Matrix Factorization
CNV: Copy Number Variation
LUAD: Lung Adenocarcinoma
LUSC: Lung Squamous Cell Carcinoma
FSQN: Feature Specific Quantile Normalization
PCA: Principal Component Analysis
MAD: Median Absolute Deviation
PAC: Proportion of Ambiguous Clustering
CDF: Cumulative Distribution Function
NSCLC: Non-small Cell Lung Cancer
GSVA: Gene Set Variation Analysis
t-SNE: T-distributed Stochastic Neighbor Embedding
AUC: The Area under the Receiver Operating Characteristic Curve
