# Supplementary figures and images for: WMRCA + : a weighted majority rule-based clustering method for cancer subtype prediction using metabolic gene sets
Source: Hereditas. 2025 Jul 7;162:121. doi: 10.1186/s41065-025-00487-4 (PMC12235908; doi:10.1186/s41065-025-00487-4)

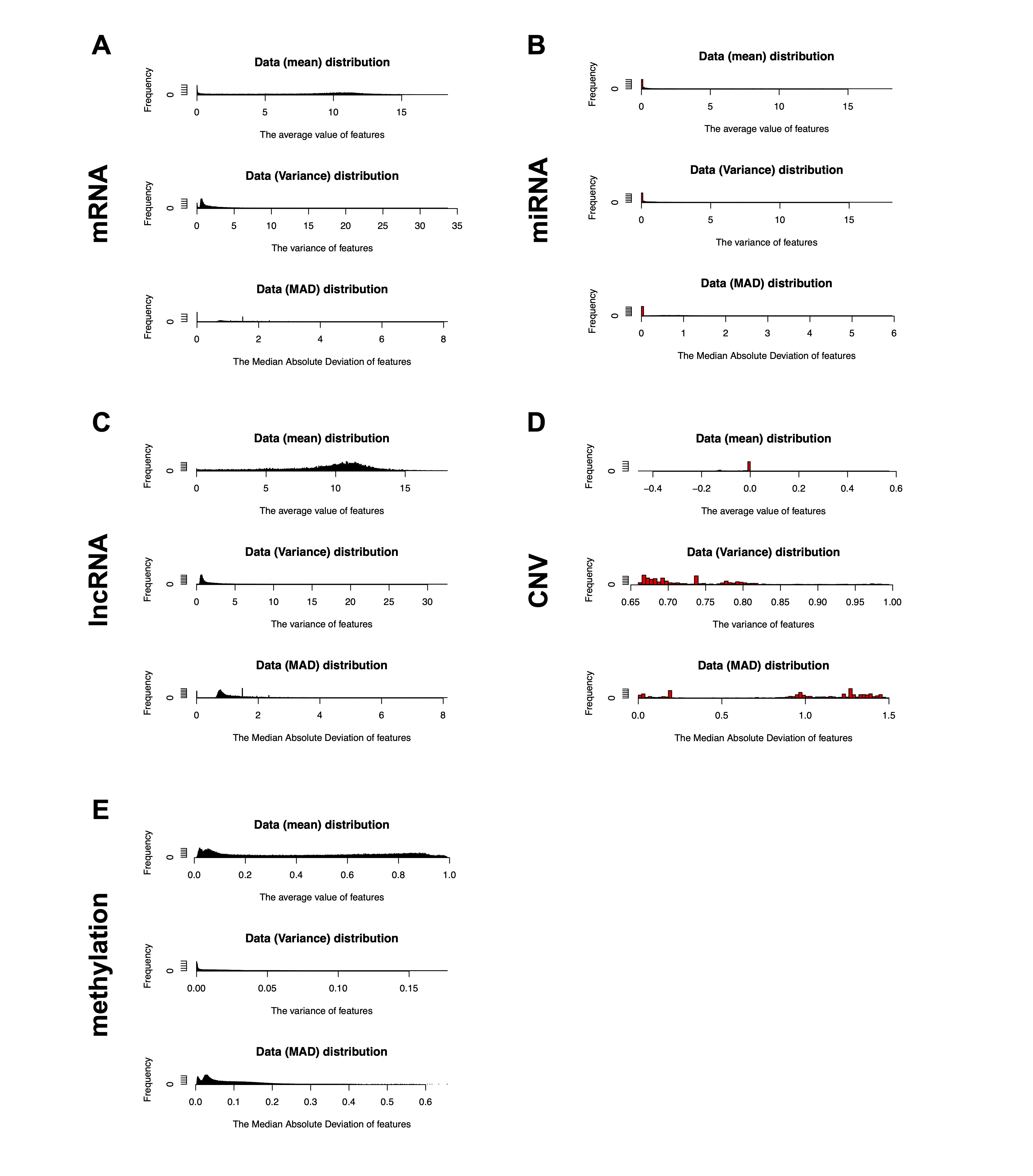

Supplement: Supplementary file 1 — Supplementary Material 1: Figure S1. Distribution of the mean, variance, and median absolute deviation of features before data filtering. [file 41065_2025_487_MOESM1_ESM.jpg]

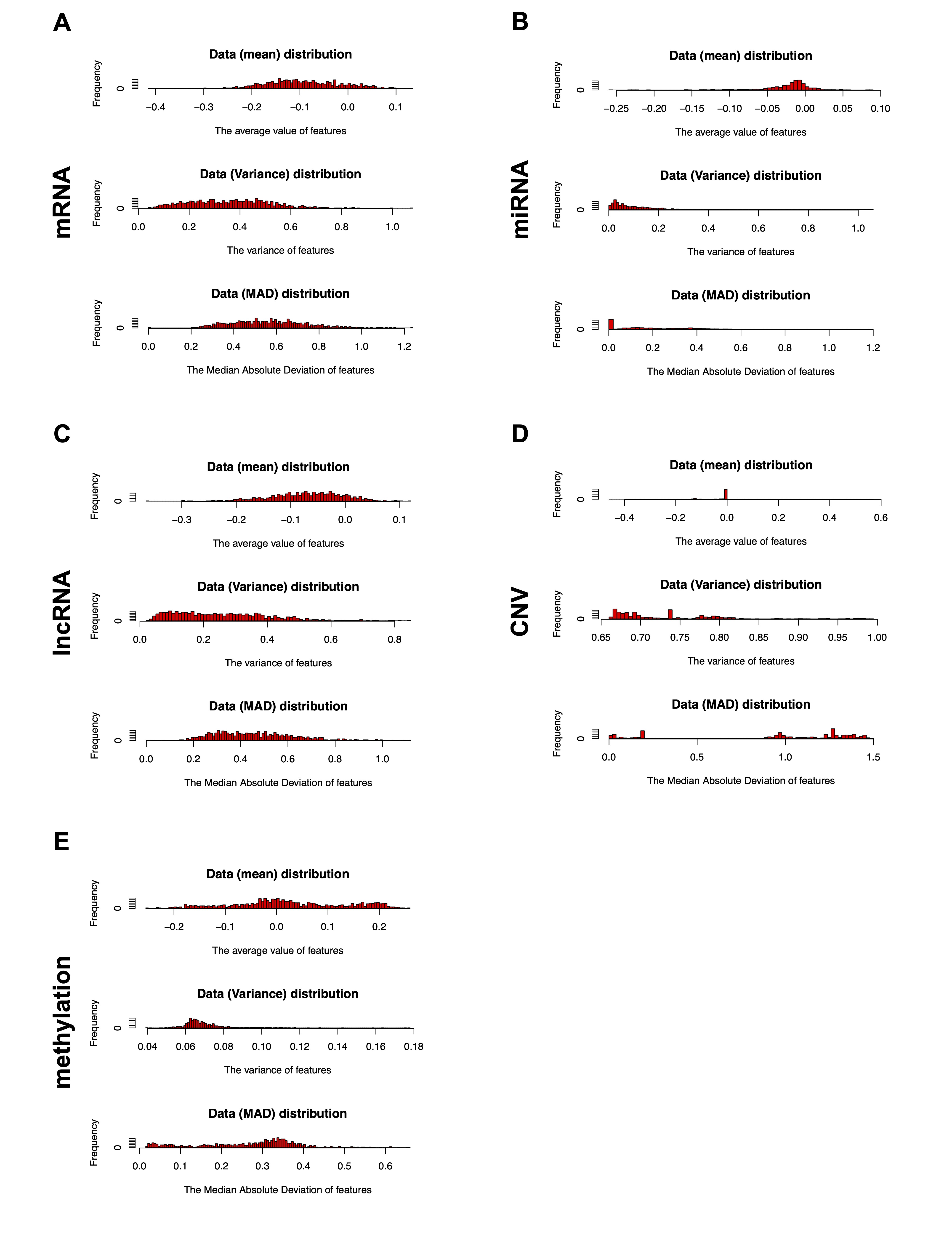

Supplement: Supplementary file 2 — Supplementary Material 2: Figure S2. Distribution of the mean, variance, and median absolute deviation of features after data filtering. [file 41065_2025_487_MOESM2_ESM.jpg]
