# Supplementary material for: WMRCA + : a weighted majority rule-based clustering method for cancer subtype prediction using metabolic gene sets
Source: Hereditas. 2025 Jul 7;162:121. doi: 10.1186/s41065-025-00487-4 (PMC12235908; doi:10.1186/s41065-025-00487-4)
Supplement: Supplementary file 3 — Supplementary Material 3: Table S1. Associations between tumor subtypes and clinical indicators based on chi-square tests. [file 41065_2025_487_MOESM3_ESM.docx]

**Table S1. Associations between tumor subtypes and clinical indicators based on chi-square tests.**

|  | ***LUAD (N=516)*** | ***LUSC (N=345)*** | ***Chi-Square*** | ***P-value*** |
| --- | --- | --- | --- | --- |
| **Pathologic T** |  |  |  |  |
| T1 | 187 (36.2%) | 88 (25.5%) | Chi-sq: 18.83, df: 4 | 0.001 |
| T2 | 277 (53.7%) | 202 (58.6%) |  |  |
| T3 | 40 (7.8%) | 44 (12.8%) |  |  |
| T4 | 8 (1.6%) | 11 (3.2%) |  |  |
| TX | 4 (0.8%) | 0 (0%) |  |  |
| **Pathologic N** |  |  |  |  |
| NA | 1 (0.2%) | 0 (0%) | Chi-sq: 14.99, df: 5 | 0.007 |
| N0 | 346 (67.1%) | 216 (62.6%) |  |  |
| N1 | 94 (18.2%) | 89 (25.8%) |  |  |
| N2 | 59 (11.4%) | 33 (9.6%) |  |  |
| N3 | 1 (0.2%) | 4 (1.2%) |  |  |
| NX | 15 (2.9%) | 3 (0.9%) |  |  |
| **Pathologic M** |  |  |  |  |
| NA | 6 (1.2%) | 2 (0.6%) | Chi-sq: 25.9, df: 3 | 0.001 |
| M0 | 314 (60.9%) | 265 (76.8%) |  |  |
| M1 | 25 (4.8%) | 5 (1.4%) |  |  |
| MX | 171 (33.1%) | 73 (21.2%) |  |  |
| **Tumor stage** |  |  |  |  |
| I | 303 (58.7%) | 163 (47.2%) | Chi-sq: 24.4, df: 4 | 0.001 |
| II | 117 (22.7%) | 117 (33.9%) |  |  |
| III | 66 (12.8%) | 55 (15.9%) |  |  |
| IV | 26 (5.0%) | 5 (1.4%) |  |  |
| NA | 4 (0.8%) | 5 (1.4%) |  |  |
| **Age at diagnosis** |  |  |  |  |
| <=65 | 246 (47.7%) | 141 (40.9%) | Chi-sq: 4.31, df: 1 | 0.029 |
| >65 | 259 (50.2%) | 201 (58.3%) |  |  |
| NA | 11 (2.1%) | 3 (0.9%) |  |  |
| **Gender** |  |  |  |  |
| Female | 281 (54.5%) | 94 (27.2%) | Chi-sq: 61.17, df: 1 | 0.001 |
| Male | 235 (45.5%) | 251 (72.8%) |  |  |
| **Number pack years smoked** |  |  |  |  |
| High | 140 (27.1%) | 169 (49.0%) | Chi-sq: 15.43, df: 1 | 0.001 |
| Low | 192 (37.2%) | 121 (35.1%) |  |  |
| NA | 184 (35.7%) | 55 (15.9%) |  |  |
| **Tobacco smoking history** |  |  |  |  |
| <=3 | 327 (63.4%) | 162 (47.0%) | Chi-sq: 25.97, df: 1 | 0.001 |
| >3 | 167 (32.4%) | 174 (50.4%) |  |  |
| Missing | 22 (4.3%) | 9 (2.6%) |  |  |
| **Primary therapy outcome success** |  |  |  |  |
| NA | 27 (5.2%) | 30 (8.7%) | Chi-sq: 13.67, df: 4 | 0.004 |
| Complete Remission/Response | 368 (71.3%) | 261 (75.7%) |  |  |
| Partial Remission/Response | 8 (1.6%) | 5 (1.4%) |  |  |
| Progressive Disease | 83 (16.1%) | 29 (8.4%) |  |  |
| Stable Disease | 30 (5.8%) | 20 (5.8%) |  |  |
| **Follow up treatment success** |  |  |  |  |
| Complete Remission/Response | 307 (59.5%) | 228 (66.1%) | Chi-sq: 7.87, df: 3 | 0.051 |
| Partial Remission/Response | 16 (3.1%) | 3 (0.9%) |  |  |
| Progressive Disease | 148 (28.7%) | 92 (26.7%) |  |  |
| Stable Disease | 45 (8.7%) | 22 (6.4%) |  |  |

*Note*：Samples with NA clinical values were excluded from the LUAD and LUSC subtypes.
